# Supplementary material for: A Molecular and Cellular Mechanism for Bitter Taste in the mosquito Aedes aegypti
Source: bioRxiv. 2026 Jul 1:2026.06.28.734668. Preprint. [Version 1] doi: 10.64898/2026.06.28.734668 (PMC13345204; doi:10.64898/2026.06.28.734668)
Supplement: Supplement 2 [file NIHPP2026.06.28.734668v1-supplement-2.pdf]

bioRxiv preprint doi: https://doi.org/10.64898/2026.06.28.734668; this version posted July 1, 2026. The copyright holder for this preprint (which was not certified by peer review) is the author/funder, who has granted bioRxiv a license to display the preprint in perpetuity. It is made available under a CC-BY 4.0 International license.

Figure S1 – expression of Gr14 in male labellum and male and female cibarium

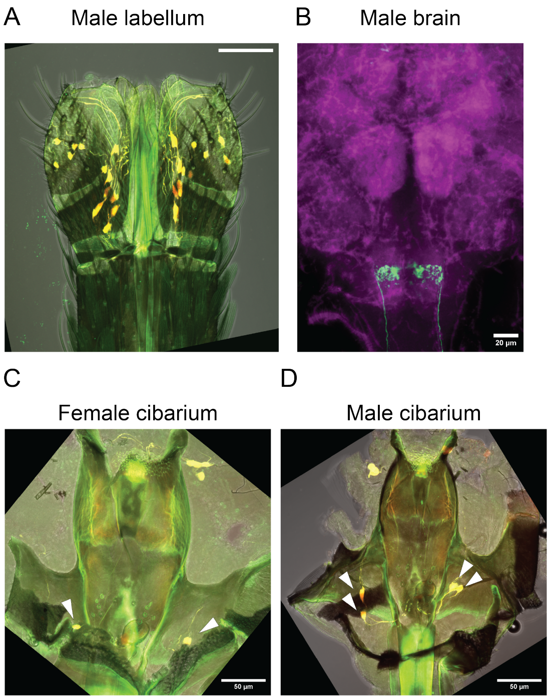

(A) Image of male labellum and labrum from *Aaeg*^{QF2} crossed to *QUAS-dTomato-T2A-GCaMP6s* (Matthews et al., 2019) shows gustatory receptor neurons (GRNs) labelled with QF2-driven fluorescence. **(E)** Immunohistochemical localization of *Gr14*-expressing GRN axons in the subesophogeal zone (SEZ) of the male brain. **(C)** Localization of *Gr14*-expressing neurons in dissected cibariums from female and **(D)** male mosquitoes. Scale bars: 50µm (A, C-D); 20µm B.

bioRxiv preprint doi: https://doi.org/10.64898/2026.06.28.734668; this version posted July 1, 2026. The copyright holder for this preprint (which was not certified by peer review) is the author/funder, who has granted bioRxiv a license to display the preprint in perpetuity. It is made available under a CC-BY 4.0 International license.

Figure S2 - Mosquitoes show neutral preference for dyes at low concentrations.

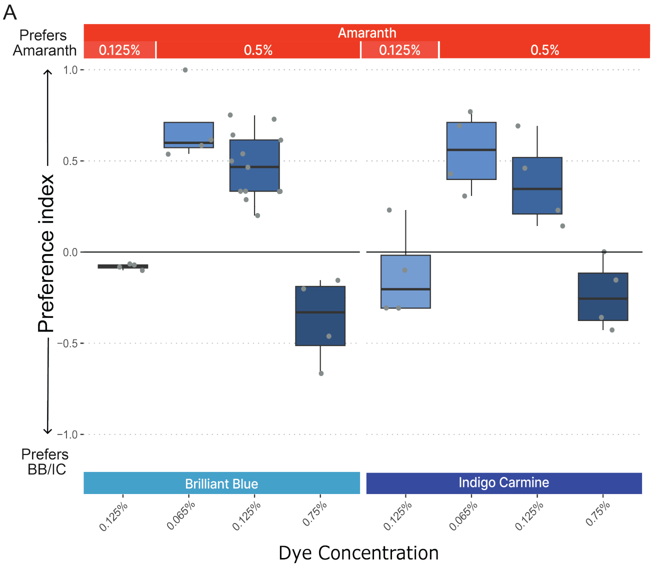

(A) The feeding preferences of female mosquitoes offered a sucrose meal with either Amaranth at varying concentrations, or Brilliant Blue (BB) dye or Indigo Carmine (IC) at varying concentrations. Feeding preference is indicated by a preference index, where 1.0 = complete preference for Amaranth dye and -1.0 = a complete preference for either Brilliant Blue dye or Indigo Carmine. Boxplots are showing the median and upper and lower quartiles. A mix of wild-type (+/+) and heterozygous (*Gr14*$^{QF2/+}$) mosquitoes were used in this experiment.

bioRxiv preprint doi: https://doi.org/10.64898/2026.06.28.734668; this version posted July 1, 2026. The copyright holder for this preprint (which was not certified by peer review) is the author/funder, who has granted bioRxiv a license to display the preprint in perpetuity. It is made available under a CC-BY 4.0 International license.

Figure S3 - Knocking out *Gr14* or silencing *Gr14* neurons does not affect feeding rates when bitters are added to a nectar meal.

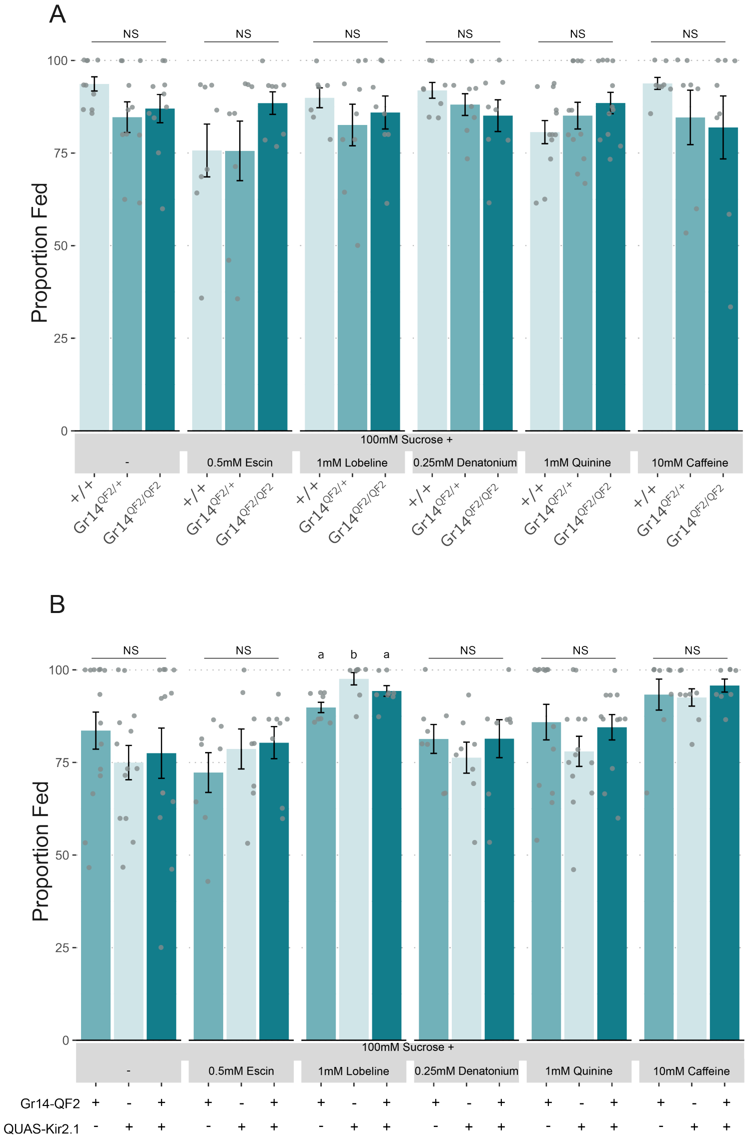

(A) Proportion of mosquitoes that fed in the mutant nectar-feeding assay for wildtype (+/+) heterozygotes (*Gr14*$^{QF2/+}$) and homoallelic Gr14 mutants (*Gr14*$^{QF2/QF2}$) across the indicated bitter treatments. **(B)** Proportion of mosquitoes that fed in the sugar-feeding assay for driver controls (*Gr14-QF2*), reporter controls (*QUAS-Kir2.1*), and Gr14-silenced mosquitoes (*Gr14 > Kir2.1*). Points represent individual cages and bars represent mean ± SEM (n = 8 trials of 9–12 mosquitoes per cage). Letters above bars indicate significant differences between genotypes within each treatment, while NS indicates no significant difference (Kruskal–Wallis test followed by a Dunn's test with a Bonferroni adjustment for multiple comparisons, $P < 0.05$).

bioRxiv preprint doi: https://doi.org/10.64898/2026.06.28.734668; this version posted July 1, 2026. The copyright holder for this preprint (which was not certified by peer review) is the author/funder, who has granted bioRxiv a license to display the preprint in perpetuity. It is made available under a CC-BY 4.0 International license.

Figure S4 - Silencing sugar neurons using Kir2.1 reduces sucrose ingestion.

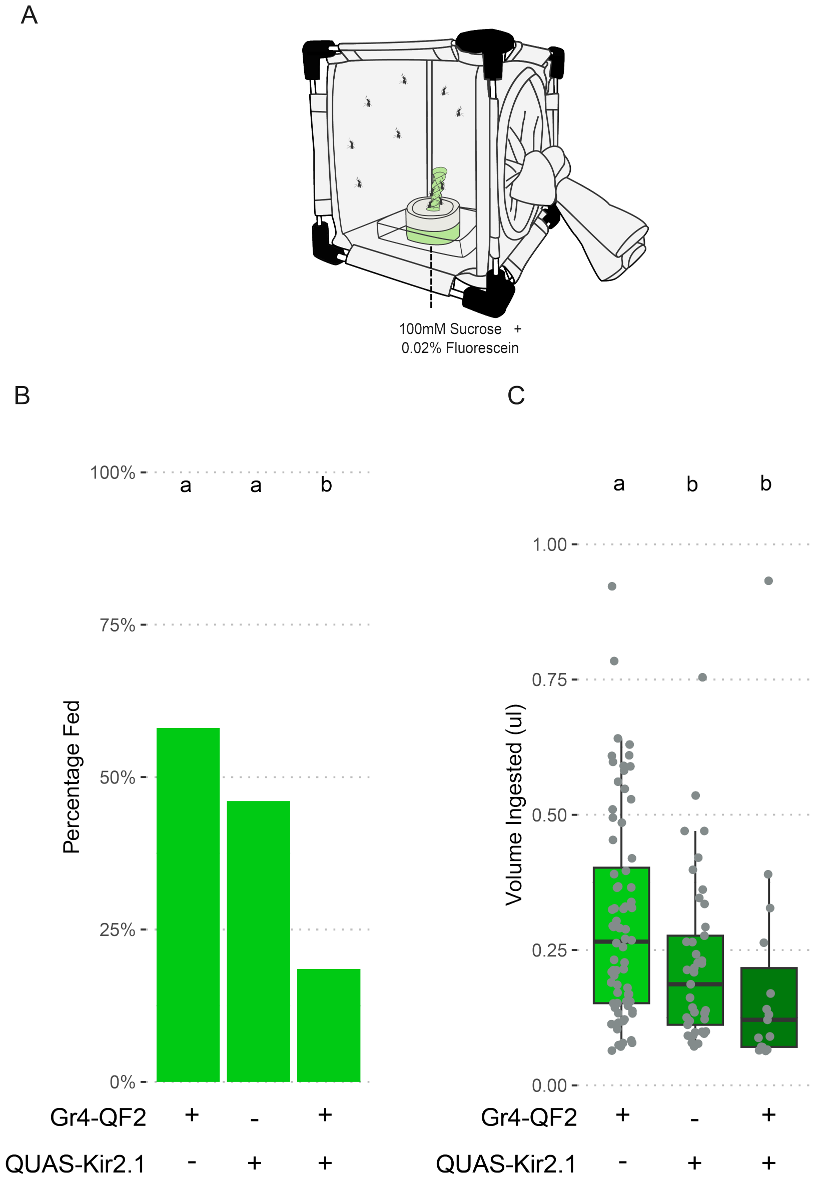

(A) Schematic of the single choice feeding assay where mosquitoes are offered a solution of 100mM sucrose, and 0.02% fluorescein **(B)** Percentage of mosquitoes that ingested 10% sucrose in 4 hours after a 24-hour water starvation. The genotypes included are driver controls (*Gr4-QF2*, n = 169), reporter controls (*QUAS-Kir2.1*, n = 116) and mosquitoes where Gr4 neurons are silenced (*Gr4 > Kir2.1*, n =111). **(B)** The volume of sucrose ingested by mosquitoes scored as fed. Letters indicate significant differences between genotypes: in **(A)** logistic regression followed by Tukey HSD, in **(B)** Kruskall-Wallis test followed by Dunn's test with a Bonferroni adjustment for multiple comparisons ($P < 0.05$).

bioRxiv preprint doi: https://doi.org/10.64898/2026.06.28.734668; this version posted July 1, 2026. The copyright holder for this preprint (which was not certified by peer review) is the author/funder, who has granted bioRxiv a license to display the preprint in perpetuity. It is made available under a CC-BY 4.0 International license.

Figure S5 – Knocking out *Gr14* or silencing *Gr14* expressing neurons does not alter feeding proportion when bitters are added directly to a bloodmeal.

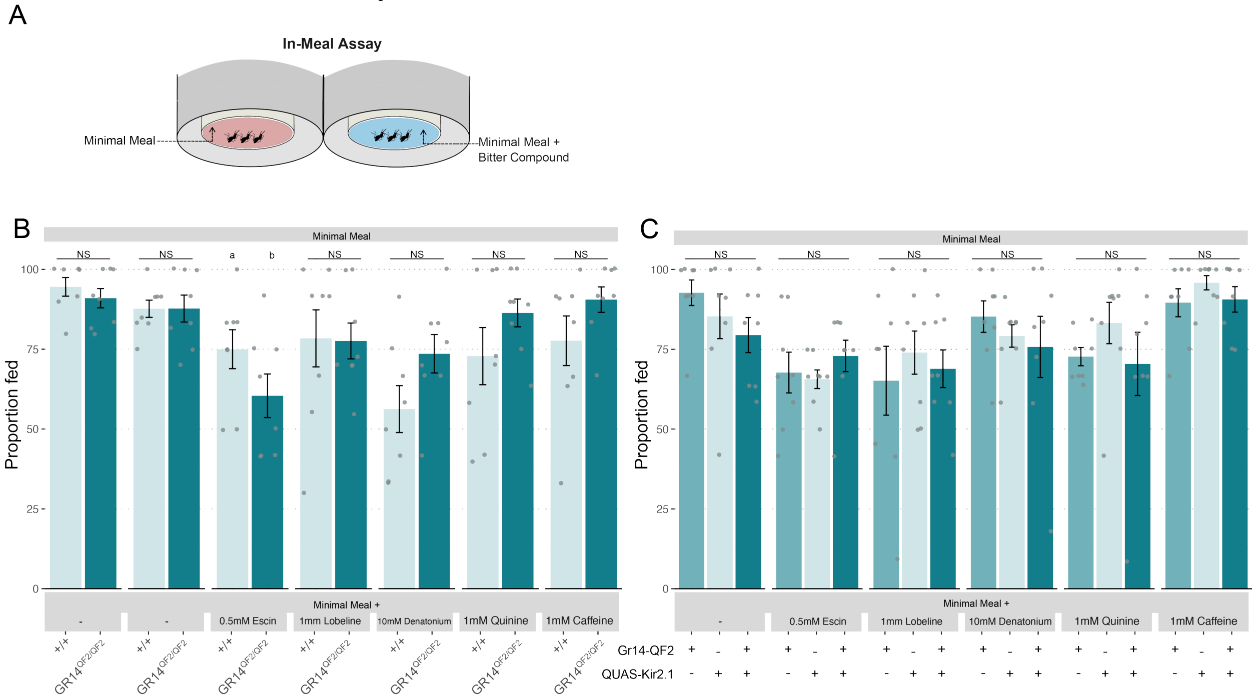

(A) Schematic of the In-Meal feeding assay, in which mosquitoes choose between a minimal meal and a minimal meal supplemented with a bitter compound. **(B)** Proportion of mosquitoes that fed in the In-Meal assay for wildtype (+/+) and homoallelic Gr14 mutants (*Gr14*$^{QF2/QF2}$) across the indicated bitter treatments. **(C)** Proportion of mosquitoes that fed in the In-Meal assay for driver controls (*Gr14-QF2*), reporter controls (*QUAS-Kir2.1*) and mosquitoes where *Gr14* expressing neurons are silenced (*Gr14* > *Kir2.1*).Points represent individual cages and bars represent mean ± SEM (n = 8 trials of 9–12 mosquitoes per cage). Letters above bars indicate significant differences between genotypes within each treatment, while NS indicates no significant difference (Kruskal–Wallis test followed by Dunn's multiple comparisons test, $P < 0.05$).

bioRxiv preprint doi: https://doi.org/10.64898/2026.06.28.734668; this version posted July 1, 2026. The copyright holder for this preprint (which was not certified by peer review) is the author/funder, who has granted bioRxiv a license to display the preprint in perpetuity. It is made available under a CC-BY 4.0 International license.

Figure S6 – Knocking out *Gr14* or silencing *Gr14* expressing neurons does not alter feeding proportion when bitters are added to the surface of a bloodmeal

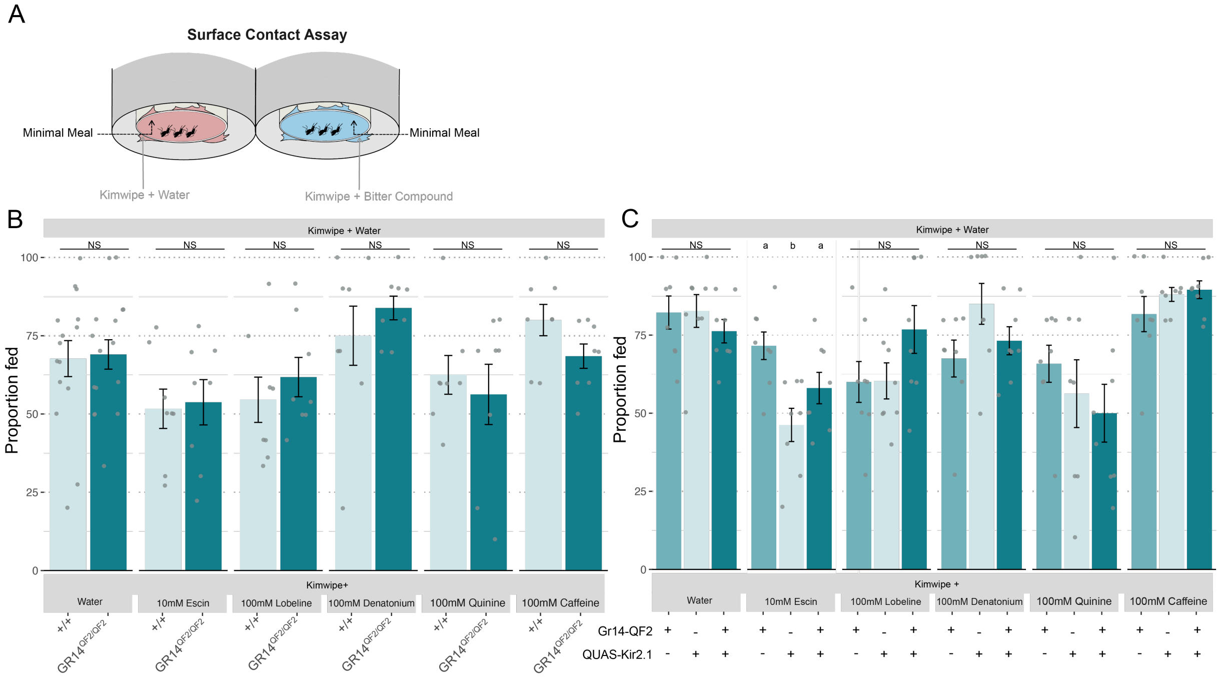

(A) Schematic of the Surface Contact assay, in which mosquitoes contact a Kimwipe treated with water or an indicated bitter compound prior to feeding on control minimal meals. **(B)** Proportion of mosquitoes that fed in the Surface Contact assay for wildtype (+/+) and homoallelic Gr14 mutants (*Gr14*$^{QF2/QF2}$). **(C)** Proportion of mosquitoes that fed in the Surface Contact assay for driver controls (*Gr14-QF2*), reporter controls (*QUAS-Kir2.1*) and mosquitoes where *Gr14* expressing neurons are silenced (*Gr14 > Kir2.1*). Points represent individual cages and bars represent mean ± SEM (n = 8 trials of 9–12 mosquitoes per cage). Letters above bars indicate significant differences between genotypes within each treatment, while NS indicates no significant difference (Kruskal–Wallis test followed by Dunn's multiple comparisons test, P < 0.05).
